# Client-therapist dyads and therapy outcome: Does sex matching matters? A cross-sectional study

**DOI:** 10.1186/s40359-022-00761-4

**Published:** 2022-03-04

**Authors:** Ileana Schmalbach, Cornelia Albani, Katja Petrowski, Elmar Brähler

**Affiliations:** 1 Department of Medical Psychology and Medical Sociology, University Medical Center of the Johannes-Gutenberg University Mainz, Mainz, Germany; 2 Abteilung Für Innere Medizin III, Universitätsklinikum Carl Gustav Carus an der Technischen Universität Dresden, Duesbergweg 6 (Campus), Dresden, Germany; 3 Department of Psychotherapy and Psychosomatic Medicine, University Hospital Leipzig, Leipzig, Germany; 4 Integrated Research and Treatment Center (IFB) Adiposity Diseases, Universitätsmedizin Leipzig, Philipp-Rosenthal-Str. 27 (Red House / House M), 04103 Leipzig, Germany; 5 Clinic and Policlinic for Psychosomatic Medicine and Psychotherapy, University Medicine Center Mainz, Mainz, Germany

## Abstract

Matching clients and therapist based on demographic variables might enhance therapeutic outcomes. Even so, research in this field is still inconclusive and not much is known about same-gender client therapist dyads in the context of cognitive behavioral (CBT) and psychodynamic methods. For this purpose, we studied the therapy outcomes of *N* = 1.212 participants that had received therapy (3 months–6 years) in Germany. The results showed a trend for same-gender client therapist dyads in terms of symptom reduction and quality of life specific to psychodynamic approaches. The latter applied specifically to female client-therapist dyads. On the other hand, this trend was not fully evident for CBT-based therapies. In conclusion, despite the robust sample and observed trends, it is not clear whether matching same gender dyads is advantageous with regards to symptom reduction and quality of life. Regardless, these results are preliminary and further studies are needed in order to find out whether same gender client-therapist dyads enhance therapy outcomes or not.

## Introduction

Identifying whether same gender client-therapist dyads enhance therapy outcomes or not, is highly relevant for clinical practice. In this way, the burden of disease for patients and health care system can be reduced. Past evidence related to client therapist gender matching lacked statistical power or analyzed a single type of disorder. Hence, we examined several diagnosis in a robust sample, which distinguishes our research from past studies. Furthermore, previous evidence did not consider gender-matching in the context of specific psychotherapy methods. Therefore, our results were examined based on two established psychotherapy methods that are covered by the German health insurance, which is key when it comes to health-associated policies or individual preferences. In addition, we illustrate a picture of the psychotherapeutic landscape in Germany from the perspective of the patients, by providing detailed information on the problems and diagnosis of patients, including their symptom development.

Matching clients and therapist based on demographic variables is common clinical practice [1], as one possible approach in trying to optimize the fit between both parties (e.g., therapeutic relationship) as well as psychotherapy outcomes [2, 3]. As suggested by past evidence, a strong therapeutic relationship predicts positive treatment outcomes [4], including positive effects in symptom reduction and general ratings of success (among others); [4–6]. Thus, it is plausible to assume that, in average, a good fit between client and therapist could be reflected in a strong bond / therapeutic relationship, as suggested by other researchers [7–9]. A good fit between clients and therapist could also refer to as having a similar understanding about managing emotions and attitudes [10–12]. Bowlby [13] reported that the psychotherapeutic relationship is comparable to the concept of attachment. Like in a parental or primary caregiver relationship, the psychotherapist offers emotional support, comfort and a “secure base”. In general, a positive therapeutic relationship is related to positive effects [4, 6, 14, 15].

Ethnicity, age or personality variables have been also used as matching indicators. Nevertheless, analyzing gender as a matching indicator is widely recommended and is one of the most examined variables in counseling research [3, 16–20]. Some researchers even discussed, that gender matched client-therapist dyads are essential for therapist to optimally adapt to the client’s needs [16, 17]. Gender dyads or matching refers to client-therapist constellations of the same gender, e.g., female clients are assigned to female therapist, while male clients will be matched to male therapist.

Theories suggest that individuals better identify and empathize with others if they believe to be similar to themselves [21, 22]. Accordingly, individuals develop certain gender-based behaviors or interactional styles and the convergence or divergence of these influences the quality of the relationship and communication with others [23–25]. In this context, gender plays an important role, since it does not only refer to physical attributes, but to cultural aspects that affect personality, attitudes, and behaviors [2, 24]. The latter affects the individuals’ world view in a way, that gendered schemas and social roles are internalized. As a result, social roles and gender expectations are reflected in specific behavioral interactional styles associated to gender [23, 25]. As an example, in western cultures men are typically socialized with traits attributed to authority and agency, such as striving for power and independence. On the other hand, women are more acquainted with communal traits or pro-social behaviors, such as solidarity and connectedness [26–28]. Correspondingly, both theories imply that same-gender client-therapist dyads have a greater convergence in terms of internalized gendered perspectives. For instance, men might instantly suppose that the male therapist will “get it” and consciously or unconsciously assume already an alliance [18]. Therefore, it is more likely that same-gender dyads share similar points of view and a comparable conceptualization of therapeutic related variables (e.g., working alliance, well-being). These are thought to account for a greater patient-therapist bond, translating into better therapeutic outcomes [3, 28].

In the case of psychotherapy, positive outcomes refer to successful treatment, measured by a favorable treatment response, i.e., reduction of disorder specific symptoms, improvement in the quality of life, lower drop-out rates and even a better working alliance [29, 30]. Many authors have posited that addressing client preferences may boost therapy outcomes. In this regard, research on client-therapist dyads has been reporting preferences towards same gender therapist [31–35]. However, in terms of outcomes empirical evidence shows inconsistent results. On the one hand, studies revealed an improvement in psychiatric symptoms of gender-matched client-therapist dyads [36, 37] reduced drop out [36, 38], better working alliance [3, 19], and greater satisfaction with the therapeutic relationship [20, 33, 36]. Importantly, a previous study demonstrated that matched clients had significantly less utilization of intensive care services, saving costs around $1000 (annually) per matched client [39]. On the other hand, authors have stated that gender matching is not a priority for clients neither an appropriate predictor of the therapy processes and outcomes [1, 40–42], especially since the reported effect sizes are small [3, 37, 43] and in some cases unknown [19, 36]. A further explanation of these mixed findings may be related to limitations in methodological procedures, small sample sizes and heterogeneity concerning type of therapy.

Until now, symptom reduction has not been well documented in the context of same gender dyads and a specific types of therapy. For example, Staczan and colleagues [20] pointed out this gap and analyzed several treatment outcomes including symptom reduction in same gender pairings. Their study showed highly significantly results in most of the studied variables in matched than in mismatched gender dyads. Nevertheless, no significant differences were shown among psychotherapy methods in terms of symptom reduction.

Regardless, the mentioned study did not examine cognitive-behavioral, behavioral, or psychoanalysis-based methods and if, some calculations included very small sample sizes (e.g., Psychodynamic *n* = 4)—making findings susceptible to random fluctuations. Therefore, it is still not clear whether gender matching is relevant or not, depending on the type of therapy patients received.

The optimization of therapeutic outcomes may persistently reduce psychological symptoms and at the same time improve the quality of life of the patients [39, 44]. Identifying whether same gender client-therapist dyads enhance therapy outcomes or not has several advantages related to enhanced therapy outcomes (e.g., better quality of life, reduction of long-term financial burden for the health care system). Since cognitive-behavioral and psychoanalysis-based (i.e., depth psychotherapeutic and psychodynamic therapy approaches) methods are covered by the German public health insurance, cost-effective measures that improve psychological interventions is a public/social concern.

Effective symptom reduction based on client therapist dyads is a very feasible procedure that can be easily implemented, if it turns out to be a useful procedure that is evidenced based. Taking the described aspects into consideration the purpose of the present study is to determine the relationship between same-gender client-therapist dyads and symptom reduction based on different types of therapies (Cognitive Behavioral Therapy, Psychodynamic approaches: e.g., Psychoanalysis and Depth psychotherapy). Based on previous findings, we expect more positive outcomes in same gender client-therapist dyads, compared to mismatched dyads. For this purpose, we assessed the following outcome variables: Symptom reduction and quality of life in two different therapy approaches, 1. CBT and 2. Psychodynamic based methods.

## Methods

### Participants

The data of the study at hand were commissioned by the University of Leipzig and approved by their ethic committee (Approval number: WREBAM16102006DGPS). The data collection was carried out by the research institute USUMA GmbH, Berlin. In general, *n* = 873 (72%) females and *n* = 339 (28%) males with different mental health conditions participated in the study—a detailed description of the sample and psychiatric disorders is displayed in Tables 1 and 2. The length of therapy that participants received according to the therapy method is presented in Table 3.

**Table 1:** Demographic variables

	Surveyed outpatient psychotherapy patients							
	Males (n = 339, 28%)	Females (n = 873, 72%)	Total (N = 1212)	German total population				
				♀ = 41.818, 51%a ♂ n = 40.184, 49%a				
Age (years)								
M	48.4	46.8	47.2					
SD	13.7	13.1	13.3					
Range	18–83	18–85	18–85					

Number (n)	Number (n)	Proportion (%)	Number (n)	Proportion (%)	Number (n)	Proportion (%)	Number (n)	Proportion (%)
18–29 years	38	11.2	97	11.1	135	11.1	9379.5	13.3b
30–44 years	85	25.1	262	30.0	347	28.6	22.353	31.5b
45–65 years	178	52.5	439	50.3	617	50.9	22.401	31.6b
Up to 65 yrs	38	11.2	75	8.6	113	9.32	16.729	23.6b
Family Status								
Married	125	36.9	345	39.5	470	38.8	35.612	50.3b
Single	117	34.5	211	24.2	328	27.1	23.063	32.5b
Legal partnership	4	1.2	3	0.3	7	0.6		
Married, living separated	15	4.4	43	4.9	58	4.8		
Widow/Divorced	78	23.0	271	31.1	349	28.8	12.187	17.2b
Divorced	60	17.7	211	24.2	271	22.4		
Widowed	18	5.3	60	6.9	78	6.4		
Highest academic degree								
Secondary school	80	23.6	162	18.6	242	20.0	28.823	42.3c
Intermediate maturity	95	28.0	339	38.9	434	35.8	19.504	28.8c
Technical / university entrance qualification	16.841	24.7c						
High School	64	18.9	140	16.1	204	16.9		
Degree	98	28.9	227	26.0	325	26.8		
Still going to school	1	0.3	2	0.2	3	0.2		
No educational qualification	1	0.3	2	0.2	3	0.2	2324	3.4c
Without information	1	0.1	567	0.8c				
Profession								
Employed	170	50.2	473	54.2	643	53.1	34.901	42.5d
Unemployed	44	13.0	76	8.7	120	9.9	4401	5.4d
Pensioner / retiree	97	28.6	186	21.3	283	23.4	20.433	24.9d
Military service / miscellaneous	12	3.5	41	4.7	53	4.4		
Not employed / housewife / husband	3	0.9	56	6.4	59	4.9		
In vocational training / pupil / student	13	4.1	41	4.7	54	4.5		
Income								
Income from relatives	22.400	27.3d						
West Germany	228	67.3	583	66.8	811	66.9	65.664	80.0e
East Germany	111	32.7	290	33.2	401	33.1	16.554	20.0e
Monthly net income / person in household (EUR)								
< 1000	138	46.8	455	59.8	593	56.2		
1000– < 1500	80	27.1	191	25.1	271	25.7		
1500– < 2000	44	14.9	90	11.8	134	12.7		
2000– < 2500	13	4.4	18	2.4	31	2.9		
2500– < 3000	10	3.4	3	0.4	13	1.2		
Ab 3000	10	3.4	4	0.5	14	1.3		

A = Microcensus 2008, total population, figures in thousands, n = 82,002,400. b = 2008 microcensus, respondents over 15 years of age, figures in thousands, n = 70,863,300. C = Microcensus2007, respondents over 20 years of age, figures in thousands, n = 68,059,000. d = Microcensus 2008, respondents over 20 years, figures in thousands, n = 82,135,000. e = Microcensus 2007, total population, figures in thousands, n = 82,218,000, no separate evidence for East and West Berlin; Berlin is one of the new federal states

**Table 2:** Conflicts and diagnosis of the patients ^a)^ and Improvement on the specified diagnosis

What complaints prompted you to seek therapeutic help?	Did the therapy helped to alleviate the symptoms / problems you sought help for?											
	Sample	Percentage	I felt much better		I felt somewhat better		Everything remained unchanged		It got worse		I am not sure/ I don’t know	
Diagnosis	n	%	%	n	%	n	%	n	%	n	%	n
Anxiety	767	63.3										
General fears	595	49.1	42.6	253	43.6	259	11.8	70	1.5	9	0.5	3
Panic attacks	333	27.5	50.6	167	31.8	105	14.8	49	2.4	8	0.3	1
Phobias	106	8.7	31.4	33	39.0	41	25.7	27	1.0	1	2.9	3
Exam anxiety	69	5.7	24.6	16	24.6	16	40.0	26	1.5	1	9.2	6
Fears of illness	252	20.8	30.2	76	39.3	99	26.6	67	3.2	8	0.8	2
Depressed or unsteady mood	1032	85.2										
Depressive complaints. e.g.. Sadness and listlessness	929	76.7	39.6	367	43.4	402	14.8	137	2.0	18	0.2	2
Bad mood. i. S. of irritability and anger	536	44.2	33.9	181	46.4	248	17.2	92	2.4	13	–	–
Grief over the loss of a loved one	394	32.5	32.4	127	40.1	157	25.0	98	1.8	7	0.8	3
Suicidality or thoughts of suicide	297	24.4	58.8	174	21.6	64	16.9	50	2.4	7	0.3	1
Addictive behavior	164	13.5										
Alcohol or drug problems	116	9.6	49.6	57	26.1	30	22.6	26	1.8	2	–	–
Other addictions (gambling, shopping, internet addiction …)	56	4.6	29.1	16	32.7	18	30.9	17	7.3	4	–	–
eating disorder	312	25.8										
anorexia	91	7.5	56.2	50	30.3	27	7.9	7	4.5	4	1.1	1
Bulimia nervosa	45	3.7	51.1	23	31.1	14	15.6	7	2.2	1	–	
Binge eating	93	7.7	33.3	31	31.2	29	33.3	31	1.1	1	1.1	1
Obesity	126	10.4	15.2	19	21.6	27	53.6	67	9.6	12	–	–
More complaints												
Compulsions/obsessions	199	16.4	41.1	81	36.5	72	16.8	33	3.5	7	2.0	4
Psychosomatic complaints	655	54.0	30.4	199	38.5	252	26.9	176	3.7	24	0.5	3
Sexual dysfunction	143	11.8	17.0	24	27.0	38	53.2	75	2.8	4	–	–
Problems coping with a physical illness	364	30.0	24.0	87	39.1	142	32.5	118	3.6	13	0.8	3
Traumatic event in life	629	51.9	30.4	191	43.9	276	23.1	145	1.7	11	1.0	6
Personality disorders	169	13.9	38.1	64	36.3	61	23.2	39	2.4	4	–	–
Other problems												
Sexual problems / conflicts	496	40.9	28.7	141	39.5	194	26.1	128	4.5	22	1.2	6
Conflicts / problems in the partnership	202	16.7	17.3	34	31.6	62	49.0	96	1.5	3	0.5	1
Problems with the children or other family members	498	41.1	36.0	166	33.0	152	26.2	121	3.3	15	1.5	7
Learning and / or work disorders	386	31.8	28.8	108	40.8	153	26.4	99	2.6	10	1.3	5
Problems in the workplace	396	32.7	30.9	105	34.7	118	27.4	93	6.2	21	0.9	3

a) = Multiple answers were possible

**Table 3:** Therapy length received by the participants in each therapy method

Psychotherapy approaches	Months *M* (*SD*)
CBT-method	13.95 (14.25)
Psychoanalysis	14.48 (16.45)
Depth-therapy	17.00 (16.55)

CBT = Cognitive Behavioral Therapy. Therapy hours were similarly distributed across therapy method

### Procedure

The data was collected in Germany in the context of a cross-sectional study design. First, in a general and nationwide telephone surveys of the population, citizens in private households who had received psychotherapy within the last 6 years or had been treated for at least 3 months were identified and asked if they were willing to provide information about their treatment. After informed consent, these participants were asked about their outpatient psychotherapy by trained interviewers in a standardized telephone interview. All households were selected via the German market research institutes (ADM) by a telephone sampling "eASYSAMPLe" (Bik-Aschpurwis and Behrend GmbH 2009) that also identifies phone numbers that are not recorded in the phone book which (Gabler-Heder method) [45]. In this way, a random selection of the households contacted could be ensured. Within the household, the target participant was also randomly determined using the “Sweden key” [46].

The inclusion criteria consisted in screening target participants of at least 18 years old, who were treated within the past 6 years or had been treated for at least 3 months. From these, *N* = 4.306 participants were targeted. A total of *N* = 1.913 (44.42%) people agreed to participate in the study. Of those who were willing to participate, only *N* = 1.212 (28.14%) interviews were carried out (response rate 74%). Reasons for exclusion of those who agreed were: The current therapy time was too short (< 3 months), the therapy was too long ago (> 6 years), the target person refused the interview (*n* = 71; 5.85%), the connection was interrupted or the person was not available (*n* = 73; 6.02%). In *n* = 170 (14.02%) cases, the interviewer found out that the target person had received physiotherapy rather than psychotherapy.

### Measures

The current study was based on questions that reflect the outpatient psychotherapeutic care in Germany from the patient's point of view, as previously reported [47]. The standardized telephone interview contained questions from "Consumer Reports" which were based on the method of Seligman's "Consumer Reports Study" [48]; German version, [49] and which was supplemented by further questions concerning the evaluation of psychotherapy. Such consisted in information about several aspects including patients’ diagnosis (e.g., anxiety disorders, depression, eating disorders), illness duration and assessment of the treatment as well as type of psychotherapy method (e.g., CBT, psychoanalysis, depth psychotherapy). For this purpose, the participants were asked: “What symptoms/complaints prompted you to seek therapeutic help?” The responses of the participants were rated by trained interviewers based on four predefined ICD diagnosis (i.e., anxiety disorders, depression, addictive behavior, eating disorders) and other somatic symptoms or complaints (e.g., coping with somatic illness, sexual problems, work related conflicts). These diagnosis and symptoms were chosen, because they are the most prevalent in Germany and most of the patients seeking psychotherapy are affected by these conditions as stated by the federal offices of statistics [50] and the society of psychiatry and psychotherapy [51].

The general state of mind of the participants was assessed at the beginning of therapy, which was reported on a 5-point scale from "1: very bad" to "5: very good". To estimate the degree of symptom reduction in the corresponding ICD diagnoses of those who completed treatment, the participants were asked: “Did the therapy helped to alleviate the symptoms / problems you sought help for?” The answers were categorized as followed: 1 = “I am doing a lot better”, 2 = “I am doing slightly better”, 3 = “… no change”, 4 = “… I am doing worse”, 5 = “…not sure/don’t know”. For the assessment of the duration of the treatment, the participants were asked to report how many sessions they had completed from the beginning until the end of treatment. A therapy session last 50 min in Germany.

For the purpose of the present study, we assessed the quality-of-life domain. Patients rated their perception in a self-report scale from 1 to 5 (1 = “I am doing a lot better”, 2 = “I am doing slightly better”, 3 = “… no change”, 4 = “… worse”, 5 = “not sure/don’t know”).

### Statistical analysis

All statistical analyses were performed with the Statistical Package for the Social Sciences (SPSS version 24.0) and R [52]. For the present study, we calculated a 2 (therapists’ sex) × 2 (patients’ sex) between ANOVA with an alpha-level of = 0.025 (Bonferroni adjustment), which takes into account multiple testing. Subsequently, post-hoc-tests (i.e., estimated marginal means and bonferroni adjusted pairwise comparisons) were computed to specify differences throughout the comparisons of dyads of the dependent variables. As an effect size we reported partial *η*^*2*^ with a 90% confidence interval. We tested the assumptions for the ANOVA (e.g., normality of residual distribution, homogeneity of variances) showing a normal distribution (*F* $\le \hspace{0.17em}$1.62, *p* ≥ 0.183). Regardless, the Shapiro Wilk was *p* < 0.001 as well as skewness and kurtosis demonstrated a light deviation (i.e., for kurtosis the largest deviation was 3.44 and for skewness 0.75). Still, the ANOVA remains robust even if the normal distribution is not given, as demonstrated by Schmider and colleagues [53].

## Results

### Therapy setting

The majority of the psychotherapist were female (57%), with a degree in Psychology (71%). Forty seven percent of the respondents mentioned behavioral therapy, 41% therapy based on depth psychology and 5% psychoanalytic therapy as a treatment method, which was implemented as individual psychotherapy in 91% of the cases. Four percent of the participants reported receiving a different psychotherapy method other than the above mentioned and 3,6% were not sure about the method received. These latter mentioned groups were not included in the analyses, since the psychotherapy method was not clear. The 698 subjects who had completed therapy had an average of 48 sessions (SD ± 68.6) and a median of 30. There were no significant differences in terms of the average treatment length across therapy methods (*F*(3, 479) = 2.43; *p* = 0.064) – see Table 3. Forty three percent of all respondents had received outpatient psychotherapy in the past. Fifty five percent of all participants took medication for their mental health condition.

### Outcomes

**Fig. 1:**
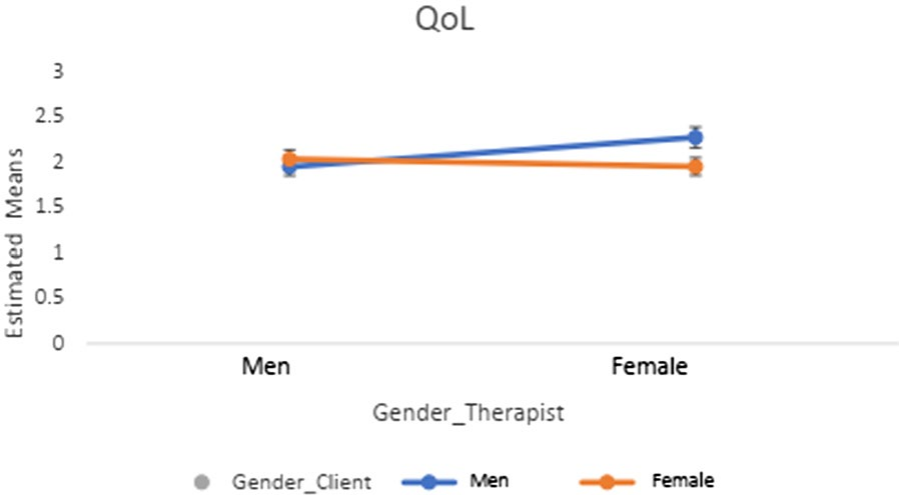
Post-hoc tests in *QoL* in psychodynamic-methods

**Fig. 2:**
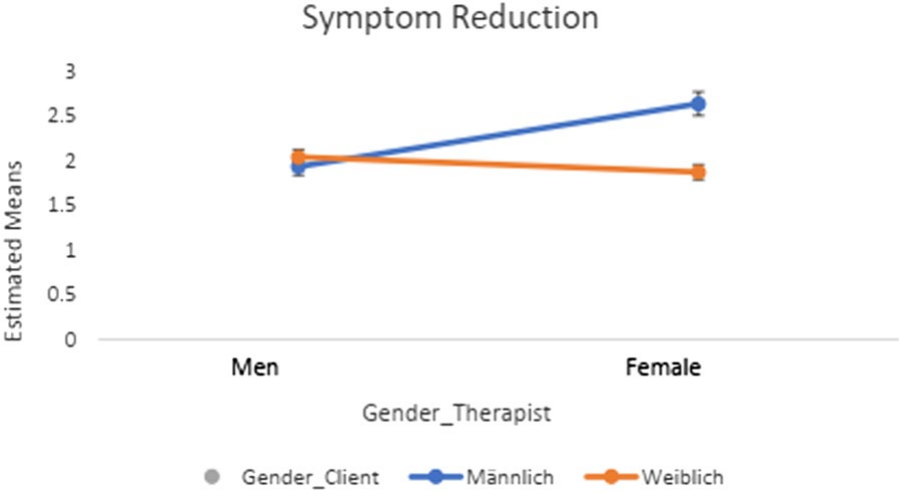
Post-hoc tests in *symptom reduction* in psychodynamic-methods

**Fig. 3:**
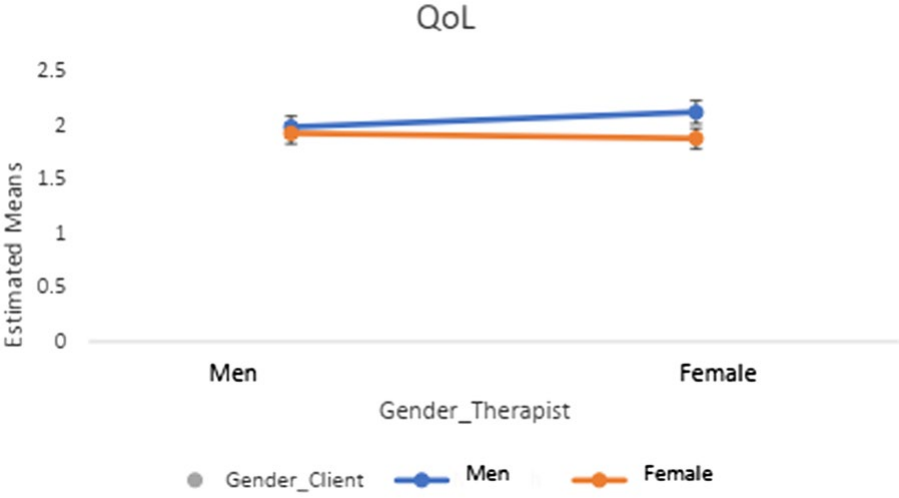
Post-hoc tests in *QoL* in CBT-methods

**Fig. 4:**
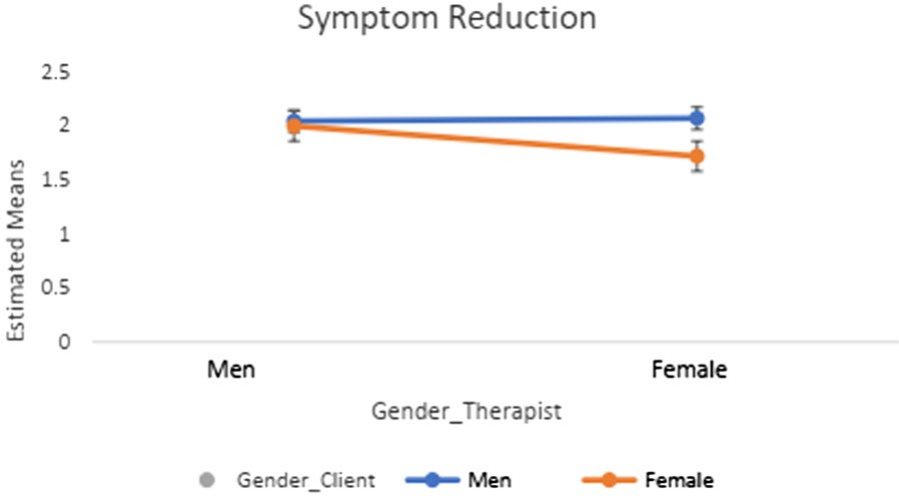
Post-hoc tests in *symptom reduction* in CBT-methods

Tables 4 and 5 show the results of the 2 × 2 ANOVA and post-hoc tests (Figs. 1, 2, 3, 4) regarding client-therapist dyads by therapy approach and examined variables (i.e., symptom reduction and QoL). Neither the gender of the client nor of the therapist indicated a significant effect for *symptom reduction* / *QoL* in the two therapy-approaches (*F* $\le \hspace{0.17em}$3.28, *p* ≥ 0.070, *η*^2^ $\le \hspace{0.17em}$0.042).

**Table 4:** Post-hoc tests and Interaction effects of gender-client therapist matching in terms of QoL and Symptom reduction in CBT

	Post-hoc-tests									Interaction effect ANOVA		
Gender therapist	Gender client		*p*	*η*^*2*^	Gender client	Gender therapist		*p*	*η*^*2*^	*F(df)*	*p*	*η*^*2*^* (CI: 90%)*
*QoL*												
Male	Male (1.98 ± .98)	Female (1.92 ± .99)	.603	.000	Male	Male (1.98 ± .08)	Female (1.92 ± .07)	.324	.002	*F*(1, 559) = 1.22	.270	.002 (0; .008)
Female	Male (2.11 ± .93)	Female (1.87 ± .80)	.047	.007	Female	Male (2.11 ± .10)	Female (1.87 ± .05)	.609	.000			
*Symptom reduction*												
Male	Male (2.05 ± 1.13)	Female (2.00 ± 1.00)	.865	.000	Male	Male (2.05 ± 1.13)	Female (2.00 ± 1.00)	.935	.000	*F*(*df*)	*p*	*η*^*2*^* (CI: 90%)*
Female	Male (2.07 ± .91)	Female (1.72 ± .76)	.224	.014	Female	Male (2.07 ± .91)	Female (1.72 ± .76)	.225	.014	*F*(1, 102) = .605	.438	.006 (0; .053)

QoL = Quality of Life; CBT = Cognitive Behavioral Method; Interaction effect = Gender client * Gender Therapist; CI = Confidence Interval

**Table 5:** Post-hoc tests and Interaction effects of gender-client therapist matching in terms of QoL and Symptom reduction in CBT

	Post-hoc-tests									Interaction effect ANOVA		
Gender Therapist	Gender client		*p*	*η*^*2*^	Gender Client	Gender Therapist		*p*	*η*^*2*^	*F(df)*	*P*	*η*^*2*^* (CI: 90%)*
*QoL*												
Male	Male (1.98 ± .98)	Female (1.92 ± .99)	.603	.000	Male	Male (1.98 ± .08)	Female (1.92 ± .07)	.324	.002	*F*(1, 559) = 1.22	.270	.002 (0; .008)
Female	Male (2.11 ± .93)	Female (1.87 ± .80)	.047	.007	Female	Male (2.11 ± .10)	Female (1.87 ± .05)	.609	.000			
*Symptom Reduction*												
Male	Male (2.05 ± 1.13)	Female (2.00 ± 1.00)	.865	.000	Male	Male (2.05 ± 1.13)	Female (2.00 ± 1.00)	.935	.000	*F*(*df*)	*p*	*η*^*2*^* (CI: 90%)*
Female	Male (2.07 ± .91)	Female (1.72 ± .76)	.224	.014	Female	Male (2.07 ± .91)	Female (1.72 ± .76)	.225	.014	*F*(1, 102) = .605	.438	.006(0; .053)

QoL = Quality of Life; CBT = Cognitive Behavioral Method; Interaction effect = Gender client * Gender Therapist; CI = Confidence Interval

Further, none of the interaction effects demonstrated a significant result (see Tables 4, 5; Figs. 1, 2, 3, 4). The results of the post-hocs test revealed the following outcomes. For CBT-methods female therapist matched with female clients reached a significantly better outcome in *QoL* compared to male therapist matched with male or female clients. Concerning psychodynamic approaches, female therapist matched with female clients reached a significantly better outcome in *QoL* compared to male therapist matched with male or female clients. Male clients matched with male therapist showed a significant improvement compared to female clients matched with male therapists. With regards to *symptom reduction*, female therapist matched with female clients obtained significantly superior results than female therapist matched with male clients (see Tables 4, 5). Overall, the post-hoc tests indicated a positive effect towards a same gender client-therapist matching especially for the female gender (vs. male client-therapist dyads) in *QoL* and *symptom reduction* within psychodynamic approaches vs. CBT-methods. For the latter, only the dyad female client and female therapist in *QoL* was significant (see Tables 4, 5).

Diagnosis of the patients and symptom reduction descriptives are illustrated in Table 2. To determine the former, the participants were asked to report their complaints and symptoms that were crucial for seeking outpatient psychotherapeutic help. The majority of the respondents reported depressive (85%) and anxiety related symptoms (63.3%), while a minority sought outpatient psychotherapy due to addictive behaviors (13.5%). Eighty four percent of the participants rated their initial condition at the beginning of therapy as “very bad” or “bad” (*M* = 1.72, *SD* ± 0.80).

Table 2 also depicts the absolute and relative frequencies of the diagnosis and complaints reported as reasons for seeking treatment as well as the distribution of the answers to the five categories to the question: “Did the therapy helped to alleviate the symptoms / problems you sought help for?” As demonstrated, most of the patients answered this question by “feeling much better” at the time they were asked to rate their perception of symptom alleviation after psychotherapy treatment. Improvement rates over 50% were observed in the following variables: Suicidality (58.8%), anorexia nervosa (56.2%) and bulimia nervosa (51.1%), panic attacks (50.6%). On the other hand, the "deterioration rates" were consistently below 5%, with the exception of “Problems in the workplace” = 6.2%.

Of those participants who completed treatment (*n* = 698, mean therapy duration 15.75 months ± SD 15.77, 48 sessions, SD ± 68.6), they experienced an improvement in their complaints and problems after an average of approx. 50–56 treatment hours. Respondents who assessed their condition as “unchanged” completed an average of 35 therapy hours.

## Discussion

The purpose of the study at hand was to determine the relationship between same-gender client-therapist dyads and therapy outcomes (e.g., symptom reduction and QoL) based on different types of therapies (i.e., CBT-Methods and psychodynamic approaches). Altogether, the main findings did not support the paradigm of improved treatment outcomes in same gender client-therapy dyads. Nonetheless, based on our analyses one could speak of a trend in favor of same gender client-therapist (female-female) in terms of *symptom reduction* and *quality of life* in the context of psychodynamic approaches, compared to CBT-based psychotherapy. This latter outcome is in line with previous research showing no effect of same gender client-therapist dyads [1, 40, 54, 55], even if not specific to CBT-based approaches. In addition, the former finding was not consistent with the literature showing a positive effect of same gender client therapist dyads on treatment outcome describing a better identification with similar others. Such understanding implies that same-gender client-therapist dyads are more likely to have a greater convergence in terms of internalized worldviews [18, 24, 25], consequently reflecting in enhanced therapy outcomes [3, 26, 28]. A possible explanation for the difference between this and our results could be possibly due to a lack of statistical power. Our study only revealed a trend in favor of female client matched with a female therapist rather than a significant result. Thus, this corresponds with the results revealed in the present study only at a descriptive level.

However, if considering the trends in the current study the better identification with similar others mostly applied to psychodynamic approaches. For the CBT-based psychotherapy *QoL* was enhanced in the female client-therapist dyad, while no other significant effect was revealed in *symptom reduction*. Concerning psychodynamic approaches, merely a trend towards an interaction effect (gender-matching) was observed.

Of greater relevance, are the significant post-hoc test revealing a female effect: i.e., mostly female therapist matched to either female or male reached significantly better outcomes in *QoL* in both therapy approaches, compared to male therapist matched to males or females. Further, *symptom reduction* was also significantly greater in female client-therapist dyads (vs. male-male dyads) if treatment was based on psychodynamic approaches. In other words, it is suggested that there is a tendency of female and male clients to benefit more from treatment provided by female therapist (vs. male therapist), as reported in the past [3, 37, 43, 56, 57]. A possible explanation could be that female therapists are more responsive and empathic towards their clients. In addition, clients of both genders also tend to respond in a more positive way to a female therapist at the beginning of the treatment, which might influence the remaining treatment course, as supported by previous evidence [56–58].

The results in psychodynamic compared to CBT can be explained by the type of therapy methodology. Psychodynamic therapies (e.g., depth psychotherapy/psychoanalysis) put more emphasis in interpersonal aspects (e.g., attitudes towards females or males), hence suggesting a greater relevance of gender [27, 28, 59], while CBT-based approaches tend to focus on modifying disorder specific behaviors. In depth psychotherapy, transference and countertransference are central aspects, whereby both, the gender of the client and the therapist may influence the therapeutic relationship [60]. For example, Tolle and Stratkötter [61] revealed that in same-gender therapy dyads transferences of both genders were reported, while in gender-mismatched dyads, the gender of the transference figure corresponded with the biological gender of the person. Nevertheless, the psychodynamic assumption of different transference patterns along gender boundaries is controversially discussed in depth psychology literature [62]. A basic assumption is that the gender of the therapist triggers (past) images in clients, which affect the interpretation of their “reality” by which transferences are build. The therapists react to this with countertransference or empathic responses, which are also influenced by gender role stereotypes [59].

An additional explanation of this female effect could be that in therapies using transference interpretations (as in the case of depth psychotherapy and psychoanalysis), women experience the relationship towards their therapist as an “affectively expressive” alliance. With male patients, female therapist might adjust the working relationship to the needs of their male clients and may grant or foster greater autonomy [63]. Additionally, it is conceivable that CBT-approaches and male-male client-therapist dyads may also fulfil the need for more distance and autonomy in male clients [63].

In spite of these findings, the therapeutic process is complex and might not be dependent on gender only. Importantly, our results are based on an observational rather than on an experimental sample and merely pointed out a trend with small to medium effect sizes. In addition, the outcomes of the present study must be interpreted with caution, since the results are based on the subjective perception of patients without considering the point of view of the therapist. Another limitation refers to the type of data collection. The results of the present study are based on a retrospective view and on a standardized interview rather than on validated instruments, making findings less comparable and perhaps less reliable. With regards to the latter concern, even if retrospective studies are a valid method to collect information, such harbor advantages and disadvantages, as every other method. For example, it is known that cognitive processes (e.g., memory) are not isolated from the current state of mind. Specifically, emotions and motivations might influence our perception and the judgments we make about the present and the past. Thus, in some cases, retrospective reports may not accurately represent specific recollections of data and rely on estimates and inferences [64, 65]. This reconstruction process could be a source of memory error, that might lead to a biased result, e.g., under or over-reporting of symptoms. Over-reporting tends to be greater for long term period events, when compared to short term periods [66]. Thus, it is possible that our participants might have overrated their symptoms, the longer ago the therapy was. Further, longer recall intervals could be associated with lower reliability of recall, and thus with a higher measurement error [65]. Even so, the recall intervals are distributed randomly across the different grouping variables. Hence, it is not expected to have adverse effects on the tests or the analyses. If anything, less reliable measurement / higher measurement error would rather lead to less statistical power and thus we would erroneously reject our hypothesis. Still, taking these aspects into consideration, further studies are needed to see how these results replicate.

Moreover, we observed a gender disproportion, especially since more female than males participated in the study. In this respect, there is also a gender imbalance with regards to the therapists, since more females compared to males participated. Thus, it could be more likely to have a higher female matching in client-therapist dyads. This situation however reflects the current occupational distribution of psychotherapist in Germany. It is estimated that around 70% of the therapist are female, with a rising trend [67]. A further limiting aspect concerns the smaller sample sizes in the variable *symptom reduction* (vs. to QoL) for both therapy methods; which compromises its representativeness. Further studies would benefit from larger samples in this domain. Perhaps future studies could target a greater responder rate by collecting data face-to-face. Possibly, an inability to create and maintain rapport vial telephone could have affected the compliance to participate, because not seeing the facial expression or body language of the interviewer might have negatively affected the response rate. Finally, our results excluded participants, who were in therapy for less than 3 months. Hence, sudden gains or worsening of symptoms in this period of time is unknown.

In sum, a remarkable strength of the present study is the large sample and the variety of disorders that patients reported, compared to past studies. Moreover, we examined most widespread psychotherapy methods covered by the health insurance in Germany, which is relevant for public health related policies, economy and for individual choices, when seeking therapy. In addition, related studies could benefit from including the perspective of the therapist in the analyses.

In conclusion, a recommendation to match same gender dyads in the context of psychotherapy is not quite clear based on our results. Therefore, more studies looking at the relationship between treatment outcome (e.g., symptom reduction in initial diagnosis) and the quality of the working alliance in the context of client-therapist gender matching with validated scales (e.g., Symptom Check-List-90, Patient Health Questionnaire, Eating Disorders Inventory, Working-Alliance, Client Attachment to Therapist Scale) are needed to shed light on the revealed trend of the present study. This could allow a clarification whether or not same gender matching is relevant, especially in the context of depth psychotherapy approaches in terms symptom reduction and quality of life. If so, the results could be useful for health care policies and for clients in terms of decision-making when seeking psychotherapy.

## Data Availability

The datasets used and/or analyzed during the current study are available from the corresponding author on reasonable request.
